# Weak signal extraction enabled by deep neural network denoising of diffraction data

**DOI:** 10.1038/s42256-024-00790-1

**Published:** 2024-02-13

**Authors:** Jens Oppliger, M. Michael Denner, Julia Küspert, Ruggero Frison, Qisi Wang, Alexander Morawietz, Oleh Ivashko, Ann-Christin Dippel, Martin von Zimmermann, Izabela Biało, Leonardo Martinelli, Benoît Fauqué, Jaewon Choi, Mirian Garcia-Fernandez, Ke-Jin Zhou, Niels Bech Christensen, Tohru Kurosawa, Naoki Momono, Migaku Oda, Fabian D. Natterer, Mark H. Fischer, Titus Neupert, Johan Chang

**Affiliations:** 1 Physik-Institut, Universität Zürich, Zurich, Switzerland; 2 Department of Physics, The Chinese University of Hong Kong, Hong Kong, China; 3 Deutsches Elektronen-Synchrotron DESY, Hamburg, Germany; 4 Faculty of Physics and Applied Computer Science, AGH University of Krakow, Krakow, Poland; 5 JEIP, USR 3573 CNRS, Collège de France, PSL University, Paris, France; 6 Diamond Light Source, Didcot, UK; 7 Department of Physics, Technical University of Denmark, Kongens Lyngby, Denmark; 8 Department of Physics, Hokkaido University, Sapporo, Japan; 9 Department of Applied Sciences, Muroran Institute of Technology, Muroran, Japan

**Keywords:** Condensed-matter physics, Computational science

## Abstract

The removal or cancellation of noise has wide-spread applications in imaging and acoustics. In applications in everyday life, such as image restoration, denoising may even include generative aspects, which are unfaithful to the ground truth. For scientific use, however, denoising must reproduce the ground truth accurately. Denoising scientific data is further challenged by unknown noise profiles. In fact, such data will often include noise from multiple distinct sources, which substantially reduces the applicability of simulation-based approaches. Here we show how scientific data can be denoised by using a deep convolutional neural network such that weak signals appear with quantitative accuracy. In particular, we study X-ray diffraction and resonant X-ray scattering data recorded on crystalline materials. We demonstrate that weak signals stemming from charge ordering, insignificant in the noisy data, become visible and accurate in the denoised data. This success is enabled by supervised training of a deep neural network with pairs of measured low- and high-noise data. We additionally show that using artificial noise does not yield such quantitatively accurate results. Our approach thus illustrates a practical strategy for noise filtering that can be applied to challenging acquisition problems.

## Abstract (web summary)

Denoising low-counting statistics data in the presence of multiple, unknown noise profiles is a challenging task in scientific applications where high accuracy is required. Oppliger and colleagues train a deep convolutional neural network on pairs of experimental low- and high-noise X-ray diffraction data and demonstrate better performance on experimental noise filtering compared with the case of training on artificial data pairs.

## Main

In recent years, remarkable progress has been made in the field of image restoration through the application of deep learning techniques^1–6^. A central task in image restoration is removing noise from an image^7–11^, where pixel *j* is composed of the intrinsic signal *s*_*j*_ and noise *n*_*j*_, *x*_*j*_ = *s*_*j*_ + *n*_*j*_. A typical benchmark problem has correlated signal between neighbouring pixels whereas the noise is uncorrelated and white. Such denoising problems have been the subject of both supervised^2,3^ and unsupervised^4,12,13^ machine-learning approaches. Supervised algorithms rely on either ground-truth (*x*_*j*_, *s*_*j*_) or noise-2-noise training pairs $({x}_{j},{x}_{j}^{\prime} )$. In the latter case, the image pairs have different (or equal) noise levels. In both cases, deep convolutional neural networks (CNNs) have been successfully applied to images with Gaussian noise^1–5^. Unsupervised approaches, sometimes dubbed noise-2-self, noise-2-void^12^ or noise-as-clean^14^, have also been employed. Their realization relies on less training information as a ground truth is absent. Unsupervised approaches therefore deliver (slightly) inferior performance compared with supervised algorithms.

Many scientific disciplines utilize digital data recording. One-, two- or three-dimensional data structures can always be transformed into a pixel-based picture format. Two-dimensional detectors are common across experimental fields such as astronomy, materials science and medical imaging. Counting of events with time-independent probability is expected to follow Poisson statistics. As such, virus–cell infection, radioactivity and particle scattering are events following a Poisson distribution. That is, the signal *s*_*j*_ and the noise *n*_*j*_ are no longer independent as the s.d. is given by ${\sigma }_{j}=\sqrt{{s}_{j}}$. Poisson noise can generally be reduced by using a sufficient acquisition time. However, long exposure times are not always possible. For example, for radiation of molecules, proteins, or human tissue, low exposure times are required to avoid beam damage^15^. Diffraction experiments in pulsed magnetic fields, by construction, have limited counting times and hence suffer from low-count (LC) statistics^16,17^. Finally, many experiments explore multi-dimensional parameter spaces that are almost impossible to cover completely with sufficient statistics. Thus, there is clear potential in developing robust methods to denoise LC statistics data to produce results of comparable quality to what would be obtained from high-count (HC) statistics data. By extension, noise filtering can speed up exploratory approaches by orders of magnitudes.

However, the removal of noise from experimental data is challenging. This can be attributed to the fact that experimental noise is the sum of multiple noise sources, such as Poisson and read-out noise, with their respective statistical properties. It is therefore difficult or often impossible to simulate experimental noise accurately. The common approach to analyse artificially added noise *n*_*j*_ to a ground-truth signal *s*_*j*_ is not directly applicable. Limited experimental training data however prevents further progress on this important problem^18,19^.

In this Article, we present experimental training data recorded by X-ray diffraction, $({x}_{j},{x}_{j}^{\prime} )=({x}_{j}^{{{{\rm{LC}}}}},{x}_{j}^{{{{\rm{HC}}}}})$, where LC and HC refer to low- and high-count statistics, respectively. Two deep CNNs were trained on such pairs to remove noise from the LC data. In a further step, the performance of the neural networks trained on experimental data was compared with the same networks trained on artificial training pairs where the HC data were corrupted with synthetic Poisson noise. We found that noise filtering of experimental noise—the ultimately relevant task—is significantly improved by training the neural networks on experimental data. This fact is particularly evident when analysing physical length scales associated with weak signals. As such, we provide a noise filtering approach for scientific data with challenging signal-to-noise features.

## Results

### X-ray diffraction data

**Fig. 1:**
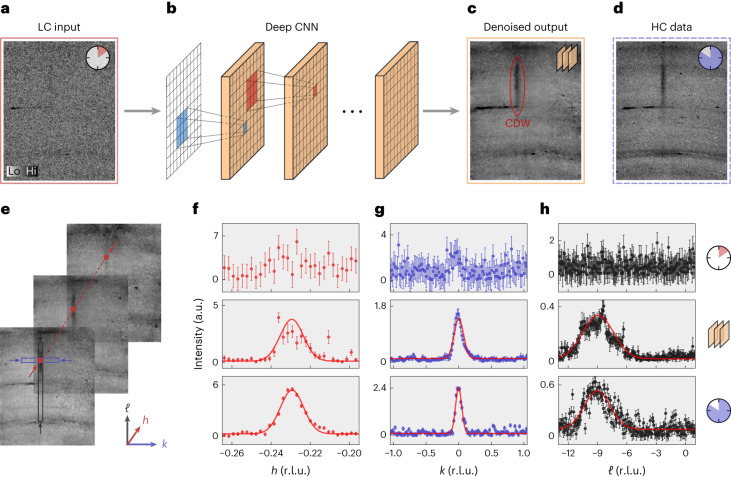
An example of denoising X-ray diffraction data using a deep CNN. **a**,**b**, A real experimental LC frame (exposure time 1 s) (**a**) is used as an input to a deep CNN (**b**) trained to remove the noise. **c**, The denoised output reveals a CDW signal (red), barely visible in the raw LC data. **d**, The real experimental HC frame (exposure time 20 s) for comparison. **e**, A stack of denoised X-ray intensity frames as in **c**. Arrows indicate the projected reciprocal coordinates *Q* = (*h*, *k*, *ℓ*). **f**–**h**, One-dimensional projected scans through *Q* ≈ (0.23, 0, 8.5) along the *h* (**f**), *k* (**g**) and *ℓ* (**h**) reciprocal space axes, in units of r.l.u. For each projected scan, a background subtraction has been performed (see main text). Gaussian fits for HC and denoised output profiles are indicated by solid red lines. The data points depicted in the denoised output profile are computed as the mean value over five training runs of the IRUNet neural network with different initial conditions. Error bars for LC and HC are shown under the assumption of counting statistics. Error bars for the denoised output are shown as the s.d. over the mentioned training runs. The clock symbols indicate relative counting time, and the network symbol indicates the denoised LC produced by the neural network.

**Fig. 2:**
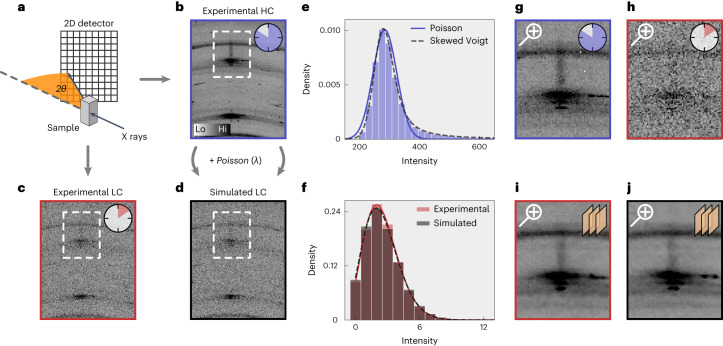
A comparison of experimental and simulated noise statistics. **a**, A schematic of the experimental X-ray diffraction set-up with scattering angle 2θ. **b**,**c**, Long exposure time leads to an HC frame (**b**), while short exposure time leads to an LC frame (**c**). **d**, A simulated LC frame obtained when adding Poisson noise to the experimental HC frame in **b**. **e**, The intensity distribution of the HC frame in **b** with fitted Poisson and skewed Voigt profiles. **f**, The intensity distribution of the experimental and simulated LC frame in **c** and **d** with a fitted Poisson profile. **g**,**h**, A zoom of the white dashed rectangular region in **b** (**g**) and **c** (**h**) encircling the CDW reflection. **i**,**j**, Zooms of the white dashed rectangular region in **c** (**i**) and **d** (**j**) after denoising using the IRUNet network trained on the respective noise distributions.

An example of X-ray diffraction intensities recorded on the high-temperature superconductor La_1.88_Sr_0.12_CuO_4_ is shown in Figs. 1a,d and 2b,c with LC and HC frames, respectively. The experimental set-up is schematically depicted in Fig. 2a and further described in Methods. Although the data cover volumes of reciprocal space, the training is carried out on two-dimensional slices (so-called frames). Therefore, the neural networks do not have access to the three-dimensionality of the data set but rely on the two-dimensional correlation of pixels in individual frames. The LC (HC) data are recorded typically for 1 s (20 s). Such experimental pairs were recorded successively with all experimental parameters fixed. The entire data set contains 7,134 frame pairs (194 × 242 pixels each) and includes signals with intensities varying over six orders of magnitude. Weak two-dimensional charge density wave (CDW) order^20–22^ manifests by vertical rod-like shapes. In cuprates, the exact nature of CDW ordering is still being debated. On an atomistic level, the CDW in La_1.88_Sr_0.12_CuO_4_ represents monoclinic distortions of the fundamental orthorhombic crystal structure^23^. Fundamental Bragg peaks (not shown) are more intense and distributed circularly over much fewer pixels. The data also contain Debye–Scherrer (powder) rings originating from the polycrystalline sample environment. Finally, the data include spurions (unidentified signal) and dead pixels. Bragg scattering implies a direct connection between scattering angle (that is, position on the detector) and incident photon energy/wavelength. As such, conclusions drawn here are invariant under different scattering angles defined by incident photon energy or sample (lattice parameter). Furthermore, a different doping concentration in our La_2−*x*_Sr_*x*_CuO_4_ show case would change the charge order incommensurability^24^ but not the overall data content.

The data set is separated into a training, validation and test set. All frames containing obvious CDW signals (our main feature of interest) are excluded from the training and validation set. These frames are instead moved to the test set, which is used for performance evaluation. Overall, the size of the training set is 3,280 pairs while the size of the validation set is 820 pairs.

### Artificial noise generation

As shown in Fig. 2f, the experimental LC data follow an approximately Poisson distribution. Therefore, to complement the experimental LC data, we artificially create LC data by adding Poisson noise to the experimental HC data. X-ray diffraction data are governed by counting statistics, where the probability of a single photon hitting pixel *j* is theoretically given by the Poisson probability distribution for large total count *N* (ideally *N* → ∞). For a fair comparison, artificial and experimental LC data should be statistically similar. To achieve this, we define *λ*_*f*_ as the ratio between the frame-integrated LC ${N}_{f}^{{{{\rm{LC}}}}}$ and HC ${N}_{f}^{{{{\rm{HC}}}}}$ (where *f* is a frame index) and *λ* = median(*λ*_*f*_). Each HC frame is then normalized with *λ* and LC frames being generated by adding the associated Poisson noise, resulting in simulated LC frames (Fig. 2d). Notice that signal intensities may vary by many orders of magnitude across the detector pixels, therefore the HC data typically display an asymmetric probability distribution (Fig. 2e).

### Deep neural network architectures and training

We implement two neural network architectures, referred to as VDSR^25^ and IRUNet^26^ (see Fig. 1a–c for a schematic illustration). The networks learn the intrinsic features of the LC input frames and produce a denoised output using the HC frames as reference. The VDSR architecture relies on stacking many convolutional layers and uses a residual learning approach to extract the noise-free data from its noisy variant^2,27^. The IRUNet architecture combines convolutional layers with an encoder/decoder framework, utilizing skip connections to reduce the vanishing gradient problem and increase accuracy. An Adam optimizer^28^ with the AMSGrad variant^29^ is used to improve convergence. All frames are normalized by their total intensity, ensuring equal scaling between LC and HC frames. During training, we apply data augmentation in the form of mirroring the frames along the *ℓ* and *k* direction and randomly adjusting the global brightness of the frames. Additional information can be found in Methods.

### Analysis

**Fig. 3:**
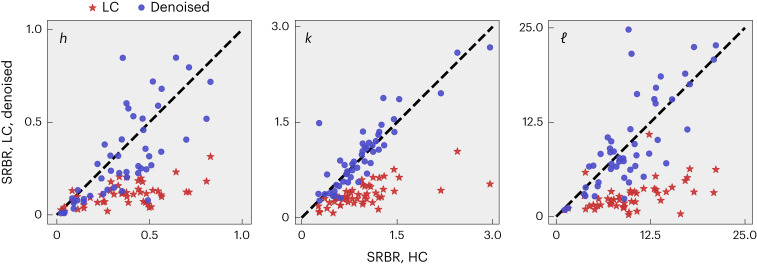
The enhancement of the SRBR when using CNN denoising via the IRUNet network trained on experimental data. Multiple frames containing CDW signals analysed along the reciprocal directions *h* (left), *k* (centre) and *ℓ* (right) in a similar fashion to Fig. 1f–h. The SRBR of the CDW reflection in the HC frame is plotted against the SRBR of the LC frame and its denoised version. We observe that the denoising of the LC frames improves the SRBR and, in many cases, even leads to better results than the HC data (scattered data points above the dashed diagonal line).

The performance evaluation of the trained neural networks (on test data) is illustrated by one-dimensional line cuts (along the reciprocal *h*, *k* and *ℓ* directions) through the CDW ordering vector (Fig. 1e–h). This involves the summation of pixel intensities within a region of interest (ROI) and subtraction of neighbouring ROIs (Supplementary Fig. 6). This subtraction is applied to eliminate the background surrounding the CDW signal, such as powder rings. As the background subtraction is not always perfect, the one-dimensional line cuts are composed of signal and a small residual ‘background’. To avoid negative residual background intensities, a small constant shift was applied. In Fig. 1f–h, we analyse the line cuts by fitting a Gaussian model. The resulting parameters are the amplitude *A*, the peak position *μ*, the s.d. *σ* and the constant residual background *C*. We furthermore define the signal to residual background ratio (SRBR) as *A*/*C*. Figure 3 shows the SRBR for 50 different examples of CDW order from the test set. Denoising using a neural network significantly improves the SRBR of the CDW order, oftentimes surpassing the results obtained from the HC data. Owing to the random nature of noise, the network is not able to learn the small but finite noise component of the HC data, resulting in efficient noise removal. These results are summarized in Table 1 in comparison with values extracted from the unfiltered LC data. We also compare the training with experimental and artificial noise of similar noise levels. Additionally to the SRBR, we calculate the mean absolute error between the denoised peak position *μ*_*h*,*k*,*ℓ*_ and the s.d. *σ*_*h*,*k*,*ℓ*_ with the HC values. From these results, we conclude that training on experimental data greatly improves the noise filtering. This conclusion holds even in the case when the amount of artificial training data is larger than the amount of experimental training data. A considerable improvement can be achieved by employing a multiscale training procedure where the artificial training data cover a wide range of statistics (Supplementary Table I). A table containing standard image quality metrics describing the denoising performance can be found in Supplementary Table III. Finally, we observe that both the IRUNet and VDSR networks, on average, achieve comparable results, despite their different architectures.

**Table 1:** Average Gaussian fitting results of different training and evaluation protocols using multiple frames from the test set containing CDW signals

	*μ*_*h*_ (×10^2^)	*μ*_*k*_ (×10^2^)	*μ*_*ℓ*_ (×10)	*σ*_*h*_ (×10^2^)	*σ*_*k*_ (×10^2^)	*σ*_*ℓ*_ (×10)	SRBR_*h*_	SRBR_*k*_	SRBR_*ℓ*_
LC	0.66 (05)	1.94 (13)	2.48 (37)	0.18 (04)	1.17 (13)	2.85 (54)	0.39 (07)	0.41 (04)	0.31 (07)
IRUNet									
Poisson → Poisson Poisson → Exp. Exp. → Exp.	0.29 (03) 0.75 (04) **0.19 (03)**	0.56 (09) 0.87 (11) **0.65 (07)**	1.08 (12) 4.17 (14) **1.47 (12)**	0.14 (02) 0.31 (04) **0.31 (03)**	0.75 (10) 1.65 (13) **0.69 (08)**	1.03 (15) 1.37 (17) **1.50 (15)**	0.90 (10) 1.02 (25) **1.41 (24)**	0.96 (03) 0.95 (13) **1.00 (02)**	1.13 (04) 0.97 (04) **1.21 (05)**
VDSR									
Poisson → Poisson Poisson → Exp. Exp. → Exp.	0.38 (02) 0.46 (03) **0.32 (02)**	0.58 (08) 0.72 (14) **0.63 (08)**	1.01 (11) 2.62 (11) **1.11 (11)**	0.14 (02) 0.19 (02) **0.16 (02)**	0.78 (09) 1.28 (18) **0.73 (08)**	1.23 (14) 1.18 (14) **0.95 (14)**	0.95 (08) 0.94 (07) **0.97 (09)**	1.05 (02) 1.15 (03) **1.09 (02)**	1.22 (05) 1.24 (05) **1.25 (04)**

The first column indicates the used training and evaluation methodology, for example, training on artificial Poisson noise and evaluation on experimental noise (Poisson → Exp.). Values given for training and evaluation on experimental noise are additionally highlighted in bold for visual guidance. The Gaussian peak position *μ*_*α*_ and s.d. *σ*_*α*_ with reciprocal space direction *α* = (*h*, *k*, *ℓ*) are given as the mean absolute error between the Gaussian parameter obtained from the denoised and the one obtained from the HC signal (the lower the better). Values for the SRBR_*α*_ are given as the absolute ratio of the Gaussian parameter obtained from the denoised and the one obtained from the HC signal (the higher the better). Values for *μ*_*α*_ as well as values for *σ*_*α*_ are scaled as indicated. Because of the broader peak in the *ℓ* direction, a scaling of 10 for *μ*_*ℓ*_ and *σ*_*ℓ*_ has been chosen over a scaling of 100.

## Discussion

The removal of experimental noise is the ultimate goal of noise filtering algorithms. Many studies have focused on filtering artificial noise from photographs^1–6^. The artificial noise typically has a single statistical distribution (Gaussian, Poisson or Bernoulli), and the photographs are represented by red–green–blue colour scales from 0 to 255. Experimental noise poses a much harder problem as multiple noise sources are present and the signal can vary by many orders of magnitude. This suggests that denoising algorithms should (also) be benchmarked on more challenging, experimental data. Here, we provide an X-ray diffraction data set where the signal intensity varies by six orders of magnitude. Not surprisingly, we find that networks trained to remove artificial noise perform well on exactly this task. However, this high performance, unfortunately, does not carry over to the filtering of experimental noise. Our results suggest that neural networks filter experimental noise better when trained on experimental noise rather than artificial noise profiles. This statement is especially true when the noise levels of the experimental and artificial noise are comparable.

**Fig. 4:**
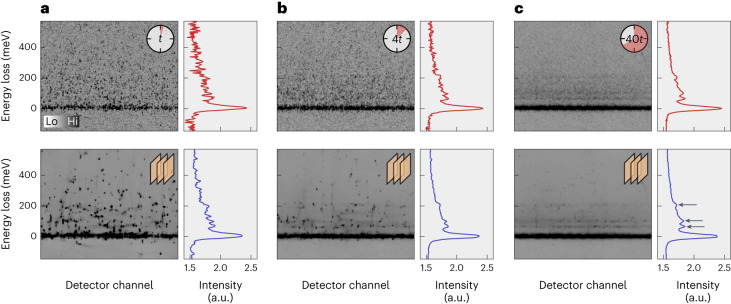
RIXS spectra recorded on SrTiO_3_. **a**–**c**, Top row: RIXS spectra with counting statistics of 1 (**a**), 4 (**b**) and 40 (**c**) times 3 min (*t*). Left: counting intensities as detector channel versus energy loss. Right: horizontally projected RIXS spectra. Bottom row: corresponding denoised neural network outputs. The arrows in **c** highlight three inelastic peaks.

To illustrate the generality and robustness of this work, we apply the trained network to resonant inelastic X-ray scattering (RIXS) data. X-ray diffraction and RIXS are fundamentally different experimental techniques (Methods). Figure 4 shows a raw RIXS spectrum recorded on SrTiO_3_ with different counting statistics as indicated (top panels). The bottom panels show the corresponding denoised output obtained from a CNN trained on experimental X-ray diffraction data exclusively. As the used RIXS detector does not offer single-photon sensitivity, the signal is not expected to follow pure Poisson statistics. Despite the dissimilar experimental technique, different sample and different noise distribution, the trained neural network achieves a visible noise reduction and consequently enhances the SRBR. The successful denoising of RIXS data likely stems from the rich variation of signals (powder rings, charge order and lattice Bragg peaks, spurious and dead pixels) and noise sources in the X-ray diffraction training data.

Our results, therefore, encourage the collection of even more diverse training data with different compositions of noise sources from other scattering, spectroscopy and microscopy techniques. Small-angle neutron and (resonant inelastic) X-ray scattering data^30^ would be obvious choices to extend the training data. Data obtained from spectroscopies^7,8^ and microscopies^9,10^ such as angle-resolved photoemission electron spectroscopy^31^ and transmission electron microscopy^32,33^ could also help expand the amount and variety of training data. Furthermore, the application of transfer learning^34^ using a pre-trained model might prove beneficial in reducing the amount of distinct training data needed. By applying our method to future studies, a large amount of beamtime could be saved, or a fixed beamtime budget could be used more efficiently by, for example, being able to probe a larger parameter space.

## Methods

### X-ray diffraction

The training data were recorded on a La_1.88_Sr_0.12_CuO_4_ single crystal^35^ at beamline P21.1 at the PETRA III storage ring at the Deutsches Elektronen-Synchrotron in Hamburg. The scattering intensities were recorded using a DECTRIS PILATUS3 X CdTe 100k detector. This detector provides 195 × 487 pixels per frame and a bit depth of 32. Each pixel is associated with a horizontal and a vertical scattering angle from which reciprocal space coordinates can be reconstructed as described in ref. ^23^. The CNN training is independent of this reconstruction that is done to extract correlation lengths. The diffractometer was operated with 100 keV photons, and the sample was cooled to around 30 K, where a CDW order is fully developed. The charge order has a short correlation length along the *c*-axis directions. Hence, along the reciprocal *c*-axis (*ℓ*), the CDW order manifests by a long rod of scattering intensity.

### RIXS

The oxygen K-edge RIXS spectra were recorded at the I21 beamline^36^ at the Diamond Light Source on a SrTiO_3_ crystal. Linear vertical light polarization and a photon energy of ~531 eV were used. The sample temperature was 20 K, and the momentum transfer was set to (*h*, *k*, *ℓ*) = (0, 0, 0.245) reciprocal lattice units (r.l.u.).

### Loss function

During each training epoch, the performance of the neural networks was determined by comparing the denoised output with the HC frame. The used loss function *L* is given by a combination of the mean absolute error and multiscale structural similarity (MS-SSIM)^7,37^. We find that this loss function results in a better overall denoising performance when compared with other losses such as the mean squared error (L2 loss) (Supplementary Fig. 1).

### CNNs

Although networks for three-dimensional data structures exist^38^, we employed architectures designed for uncorrelated two-dimensional images. A comprehensive review of deep learning and CNNs applied to noise filtering of images is given in ref. ^11^. Generally, many networks display comparable performance. In this work, we implemented two different neural network architectures, referred to as VDSR^25^ and IRUNet^26^. For the VDSR architecture, we did not include the final addition layer as we do not find a significant performance change (Supplementary Table II). The weights of the convolutional layers are randomly initialized using the He method^39^. For the VDSR model, we make use of a parametric rectifying linear unit^39^ after each convolutional layer, while a normal rectifying linear unit is used in the IRUNet architecture. The VDSR network was trained for 150 epochs using a batch size of 8 and an initial learning rate of 5 × 10^−4^. The IRUNet network was trained for 200 epochs using a batch size of 16 and an initial learning rate of 5 × 10^−4^. The learning rate was decreased after a certain number of epochs to ensure good convergence. For the VDSR model, we multiplied the learning rate by 0.5 after every 50 epochs. For the IRUNet model, the learning rate was multiplied by 0.5 after 150 epochs. The total training duration of the VDSR and IRUNet models was on average around 20 and 10 h, respectively, on an Nvidia Tesla P100 GPU with 10 GB of VRAM using TensorFlow 2.4.1. A discussion of the receptive field of the neural networks can be found in Supplementary Section G.

### Supplementary information

Supplementary Information Supplementary Discussion, Figs. 1–6 and Tables 1–3.

## Data Availability

The experimental data used in this work can be found at 10.5281/zenodo.8237173 (ref. ^40^).

## Code availability

The code used for the set-up and training of the neural networks is available at 10.5281/zenodo.10245374 (ref. ^41^).
