# Supplementary material for: Weak signal extraction enabled by deep neural network denoising of diffraction data
Source: Nat Mach Intell. 2024 Feb 13;6(2):180–6. doi: 10.1038/s42256-024-00790-1 (PMC10883886; doi:10.1038/s42256-024-00790-1)
Supplement: Supplementary file 1 — Supplementary Discussion, Figs. 1–6 and Tables 1–3. [file 42256_2024_790_MOESM1_ESM.pdf]

nature machine intelligence

Article https://doi.org/10.1038/s42256-024-00790-1

Weak signal extraction enabled by deep neural network denoising of diffraction data

In the format provided by the authors and unedited

CONTENTS

A. Details about the loss function 2
B. Comparison of CNN-based noise filtering and conventional denoising (smoothing) 2
C. Overfitting 3
D. More artificial training data, multiscale training, and network modifications 3
E. Evaluation using standard image quality metrics 4
F. Influence of training data shuffling, random seed, and optimizer 5
G. Receptive field of the neural networks 6
H. Background subtraction 7

References 7

A. Details about the loss function

In this work we made use of a loss function that is known to perform well for images intended for evaluation by a human observer [1, 2]. It combines a pixel-wise absolute error (mean absolute error, MAE) with local structural similarity that is calculated at different scales (multiscale structural similarity, MS-SSIM [3, 4])

$$L = (1-\alpha)L_{\text{MAE}} + \alpha L_{\text{MS-SIM}} \quad \text{with} \quad \alpha = 0.7$$

where the value of α has been chosen empirically. In Figure 1 we show the denoising performance on a single low-count (LC) frame when using aforementioned and other standard loss functions such as mean absolute error (MAE, L1) and mean squared error (MSE, L2). We observe that the MSE loss leads to poor performance, resulting in a locally smeared background with only minimal structural patterns. MAE produces a more even background but fails to faithfully represent the faint charge-density-wave (CDW) signal. Combining MAE with MS-SSIM shows a background that is more consistent with the one of the high-count (HC) frame. Additionally, structural patterns including the CDW signal are clearly enhanced.

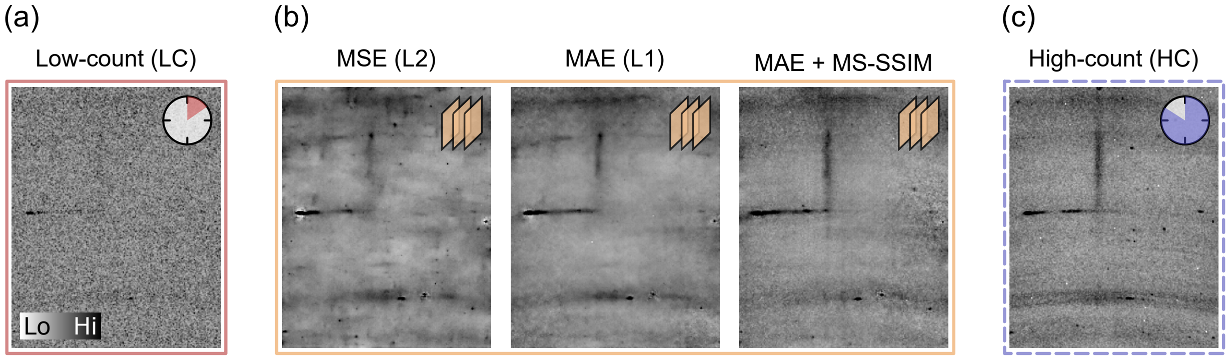

FIG. 1. Impact of different loss functions on the denoised neural-network output. (a) Low-count frame. (b) Denoised low-count frame in (a) using different loss functions during the training of the network. A combination of mean absolute error and multiscale structural similarity (MAE + MS-SSIM) shows the best denoising performance. (c) High-count frame for comparison.

B. Comparison of CNN-based noise filtering and conventional denoising (smoothing)

A commonly used practice to reduce the noise in (2D) data is smoothing. One example is Gaussian smoothing where the noisy data is convoluted with a Gaussian kernel of a certain standard deviation. In Figure 2 we compare a conventional Gaussian smoothing approach and CNN-based noise filtering. While the Gaussian smoothed low-count (LC) frame results in a reduction of the high-frequency noise, it inevitably blurs the data [2]. On the contrary, the LC frame produced by the trained CNN effectively suppresses the high-frequency noise present in the LC data while the weak CDW signal – barely visible in the LC frame – is strongly enhanced. This is achieved by a significant improvement in the local signal continuity following the application of the CNN-based noise filtering.

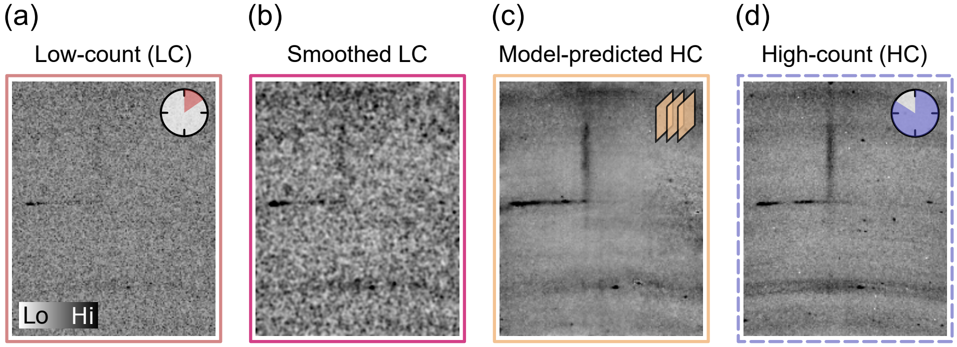

FIG. 2. Comparison of CNN-based denoising and conventional Gaussian smoothing. (a) Low-count (LC) frame. (b) Gaussian smoothed low-count frame in (a) using a standard deviation of 1. (c) Denoised low-count frame in (a) using a trained CNN. (d) High-count (HC) frame for comparison.

C. Overfitting

During the training process, we evaluate the neural-network performance using a separate validation data set as mentioned in the main text. We keep track of the resulting validation loss to ensure optimal convergence without overfitting, which shows itself in an increase of the validation loss. It implies that the model cannot perform well on unseen data, as it might have overly specialized in learning the features of the training data. In Figure 3(a) we compare the loss curves for the two used neural network architectures, IRUNet [5] and VDSR [6]. In Figure 3(b), loss curves for different training data statistics are shown. Figure 3(c,d) shows the effect of simulated counting statistics, which will be further elaborated in the next section.

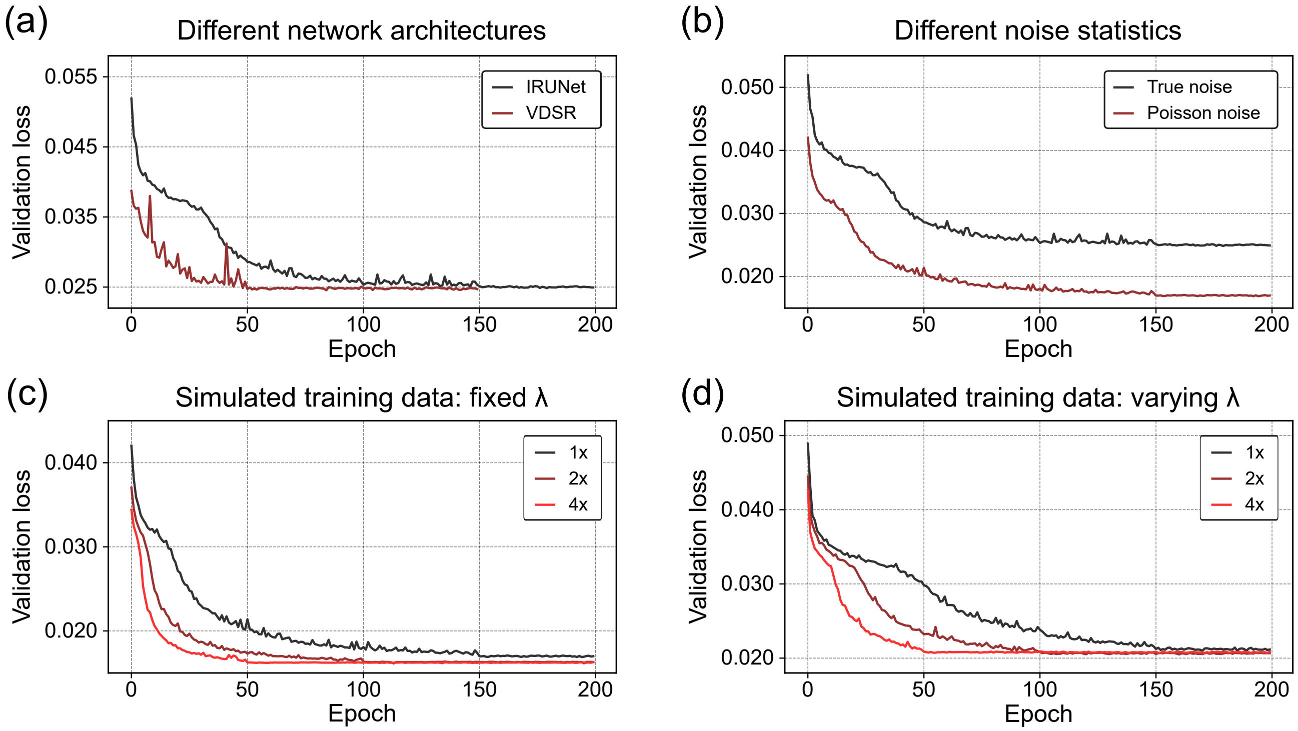

FIG. 3. Loss curves showing training epoch against validation loss for different training scenarios. (a) Different neural-network architectures. (b) Training on different noise statistics (experimental or simulated Poisson noise). (c) Simulating artificial low-count images with the same Poisson statistics (fixed λ) for different amounts of artificial training data. For example 2x refers to double the amount with respect to the size of the original training data set. (d) Simulating artificial low-count images with varying λ (multiscale training) for different amounts of artificial training data.

D. More artificial training data, multiscale training, and network modifications

One of the advantages of artificial training data is the fact that one can simulate an arbitrary amount of low-count (LC) frames given a single high-count (HC) frame by drawing random samples from the underlying Poisson distribution with a fixed λ factor as described in the main text. Increasing the amount of training data is a well-known approach to enhance the performance of neural networks. Additionally, the used experimental X-ray diffraction training data consists of LC (HC) pairs with exposure times of 1 (20) seconds for the most part ($> 80\%$). However, there are some cases where the frames have been counted for slightly different times, which effectively changes the λ factor of the Poisson distribution. In those cases where the statistics differ throughout the test data set, one should benefit from a multiscale (MS) training approach where the network is trained on LC data with different counting times (varying λ) [2, 7]. Both of these potential improvements regarding the training with artificial training data have been studied. For training a MS model we choose a uniform distribution of 100 λ values $\in [0.001, 0.1]$. The convergence of the validation loss for these training approaches is shown in Figure 3(c,d) where the learning rate has been reduced by half after the first 150, 100, and 50 epochs for 1x, 2x, and 4x amount of training data respectively. We note that the final validation loss does not change that much. However, the amount of required training epochs for convergence is strongly reduced when using more training data. Doubling the amount of training data will roughly

half the amount of required epochs. We would like to point out that while the amount of epochs is reduced, the overall training time does in fact not decrease but rather scales with the amount of training data. The training time required for convergence is around 7, 11, and 16 hours for 1x, 2x, and 4x data respectively using an Nvidia Tesla P100 GPU with 10 GB of VRAM. The performance results of mentioned training approaches are summarised in Table I using the IRUNet architecture and 50 different CDW signals as described in the main text. We observe that additional artificial training data with the same statistics (fixed λ) is still inferior to training with real experimental data. We furthermore observe that employing a multiscale approach does benefit training with artificial noise, sometimes even attaining comparable performance to training with actual experimental data. A multiscale approach could therefore prove valuable when dealing with only a limited amount of experimental training data, provided that the underlying statistics are well-known.

Additionally, we have implemented a VDSR network architecture [6] without the final residual layer. We find that removing said layer doesn't lead to a significant change in performance as shown in Table II and III.

TABLE I. Average Gaussian fitting results of different training scenarios using the IRUNet network architecture. The first column indicates the amount of used (artificial) training data and whether a multiscale (MS) procedure has been utilized. For example, using double the amount of artificial training data and applying a multiscale procedure (2x Poisson MS). The results when training on the original amount of experimental training data are shown at the bottom for comparison (Exp. → Exp.) and are additionally highlighted in bold for visual guidance.

	μ_h ($\times 10^2$)	μ_k ($\times 10^2$)	μ_ℓ ($\times 10$)	σ_h ($\times 10^2$)	σ_k ($\times 10^2$)	σ_ℓ ($\times 10$)	SRBR_h	SRBR_k	SRBR_ℓ
1x Poisson	0.75 (04)	0.87 (11)	4.17 (14)	0.31 (04)	1.65 (13)	1.37 (17)	1.02 (25)	0.95 (13)	0.97 (04)
2x Poisson	0.66 (35)	1.68 (14)	3.11 (13)	0.39 (35)	1.53 (11)	1.55 (15)	0.87 (15)	1.04 (34)	0.95 (04)
4x Poisson	0.44 (02)	2.00 (10)	3.24 (15)	0.16 (02)	0.94 (15)	1.28 (17)	0.88 (08)	0.98 (06)	1.04 (04)
1x Poisson MS	0.32 (07)	0.73 (07)	1.23 (12)	0.33 (07)	0.80 (08)	1.10 (15)	0.92 (13)	1.13 (02)	1.11 (04)
2x Poisson MS	0.35 (02)	0.69 (07)	1.08 (12)	0.18 (02)	0.66 (07)	1.01 (15)	0.89 (08)	1.06 (02)	1.09 (04)
4x Poisson MS	0.45 (02)	0.65 (07)	2.66 (13)	0.19 (02)	0.72 (07)	1.22 (16)	0.91 (07)	1.06 (02)	1.15 (05)
Exp. → Exp.	**0.19 (03)**	**0.65 (07)**	**1.47 (12)**	**0.31 (03)**	**0.69 (08)**	**1.50 (15)**	**1.41 (24)**	**1.00 (02)**	**1.21 (05)**

TABLE II. Average Gaussian fitting results for the VDSR network architecture with (⊕) and without a final residual layer, trained and evaluated on experimental data.

	μ_h ($\times 10^2$)	μ_k ($\times 10^2$)	μ_ℓ ($\times 10$)	σ_h ($\times 10^2$)	σ_k ($\times 10^2$)	σ_ℓ ($\times 10$)	SRBR_h	SRBR_k	SRBR_ℓ
VDSR	0.32 (02)	0.63 (08)	1.11 (11)	0.16 (02)	0.73 (08)	0.95 (14)	0.97 (09)	1.09 (02)	1.25 (04)
VDSR (⊕)	0.20 (10)	0.63 (07)	1.31 (01)	0.14 (01)	0.64 (08)	0.81 (14)	1.00 (11)	1.13 (02)	1.47 (05)

E. Evaluation using standard image quality metrics

In this study, we assessed the denoising performance of the trained neural networks based on physical signal properties, including the signal-to-residual-background ratio (SRBR) of the CDW peak. Such an evaluation removes potential ambiguities that might emerge when using standard image quality metrics, such as peak signal-to-noise ratio (PSNR) or structural similiarty (SSIM) [3, 4], as these metrics necessitate a noise-free ground truth image. However, given the nature of experimental data, a finite amount of noise persists even with high counting statistics. This inherent noise renders an evaluation purely based on mentioned image quality metrics less favorable. For completeness, those metrics are summarized in Table III next to unambiguous mean absolute (MAE) and mean squared errors (MSE).

TABLE III. Denoising performance using standard image quality metrics for different neural-network architectures and training scenarios. The evaluation has been performed on true experimental data from the separate test set mentioned in the main text. The values are given as the mean (median) over all test images. The first column indicates the used network architecture. The second column refers to the amount of used (artificial) training data and whether a multiscale (MS) procedure has been utilized. For example, using double the amount of artificial training data and applying a multiscale procedure (2x Poisson MS). The results when training on the original amount of experimental training data are shown at the bottom of each network section for comparison (Exp. → Exp.) and are additionally highlighted in bold for visual guidance.

		MAE - L1 ($\times 10$)	MSE - L2 ($\times 10^2$)	PSNR (dB)	MS-SSIM
IRUNet	1x Poisson	0.153 (0.109)	0.073 (0.021)	35.510 (36.838)	0.952 (0.977)
	2x Poisson	0.141 (0.108)	0.059 (0.020)	35.766 (36.995)	0.953 (0.978)
	4x Poisson	0.138 (0.107)	0.049 (0.020)	35.871 (36.999)	0.962 (0.978)
	1x Poisson MS	0.122 (0.118)	0.027 (0.024)	35.819 (36.178)	0.974 (0.977)
	2x Poisson MS	0.115 (0.112)	0.025 (0.022)	36.259 (36.638)	0.975 (0.978)
	4x Poisson MS	0.117 (0.113)	0.025 (0.022)	36.185 (36.564)	0.975 (0.977)
	Exp. → Exp.	**0.118 (0.111)**	**0.028 (0.022)**	**36.00 (36.530)**	**0.973 (0.977)**
VDSR	1x Poisson	0.123 (0.114)	0.032 (0.022)	35.744 (36.501)	0.965 (0.975)
	Exp. → Exp.	**0.116 (0.111)**	**0.027 (0.022)**	**36.011 (36.506)**	**0.973 (0.976)**
VDSR (⊕)	1x Poisson	0.127 (0.114)	0.034 (0.022)	35.794 (36.544)	0.965 (0.976)
	Exp. → Exp.	0.127 (0.121)	0.030 (0.025)	35.540 (35.973)	0.971 (0.973)

F. Influence of training data shuffling, random seed, and optimizer

A common technique to evaluate the performance of a trained machine-learning model is k-fold cross-validation [8] where the entire data set is split into k equally-sized parts. One part is used for validation while the remaining $k-1$ parts are combined into the training data set. As described in the main text, we use a 4:1 splitting ratio for training and validation data set (3280 training and 820 validation pairs). In Figure 4(a) we show the result of cross-validation for $k = 5$ splits. In particular, we observe that the final loss and denoising performance is independent of the chosen training-validation splitting.

In general, the chosen random seed, used for the initialization of the network parameters, can significantly influence the final performance when training deep-learning models [9, 10]. It is thus advised to verify that training results are reproducible. As such, we conducted an experiment where we varied the random seed – see Figure 4(b). While we observe slightly larger fluctuations of the loss curve and image quality metrics compared to Figure 4(a), the final performance does not appear to be strongly affected.

Finally, an adaptive momentum estimation (Adam) optimizer [11] was used for training the deep neural networks in this work. However, it has been reported that stochastic gradient descent (SGD) methods are better at generalizing and finding a broader global optimum [12, 13]. In Figure 5(a) we compare loss curves of Adam, SGD, and stochastic weight averaging (SWA) [14] while in Figure 5(b) we show their denoising performance using standard image quality metrics. For SGD and SWA, variants with and without momentum have been considered. As described in the methods section of the main text, a learning rate of 5×10^{-4} has been used for Adam while SGD and SWA runs were performed using a learning rate of 1×10^{-1}. These learning rates have been found to yield the best final validation loss for the respective optimizers over 200 epochs using the IRUNet architecture. For the last 50 epochs the learning rate has been reduced by half. A momentum of 0.9 has been chosen for the momentum-variants of SGD (SGDm) and SWA (SWAm). Overall, we find that Adam results in a considerably better denoising performance compared to SGD and SWA despite the fact that it has a larger spread (error bars in Figure 5(b)). We also observe that SGD (SWA) with momentum tend to yield slightly better results compared to their non-momentum counterparts.

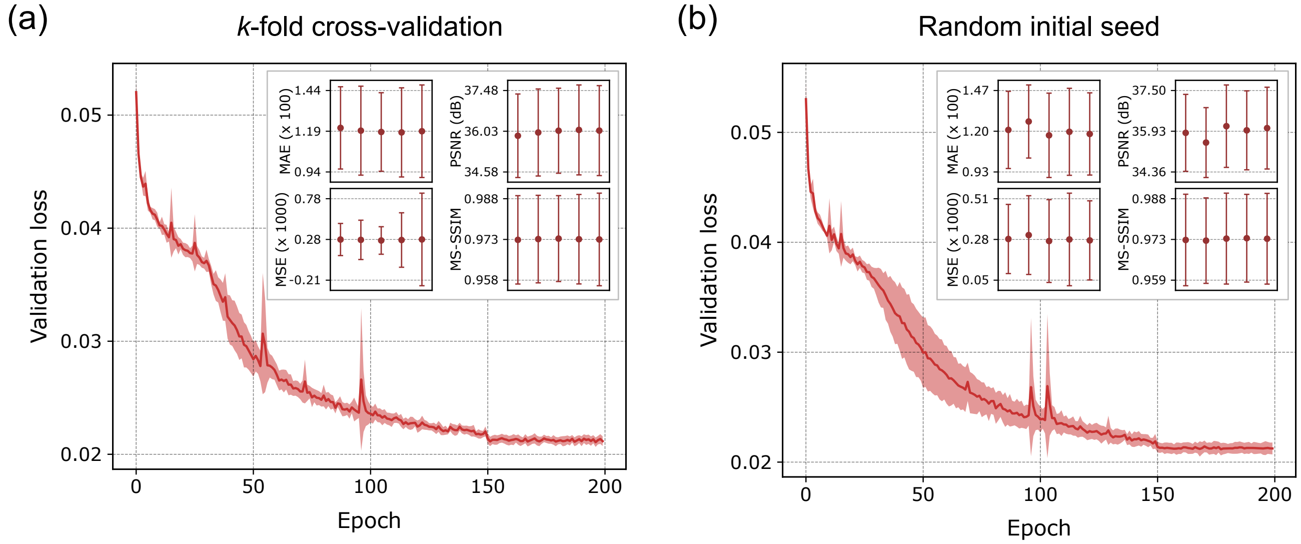

FIG. 4. Denoising performance for (a) k-fold cross-validation ($k = 5$) and (b) different random seeds (5) for the initialization of the network weights using the IRUNet architecture. The validation loss curve corresponds to the mean, while the shaded area corresponds to the standard deviation of the validation loss over the performed training runs. The insets in (a) and (b) show mean values (dots) and standard deviation (error bars) of standard image quality metrics obtained after evaluating the trained networks on the separate test set described in the main text.

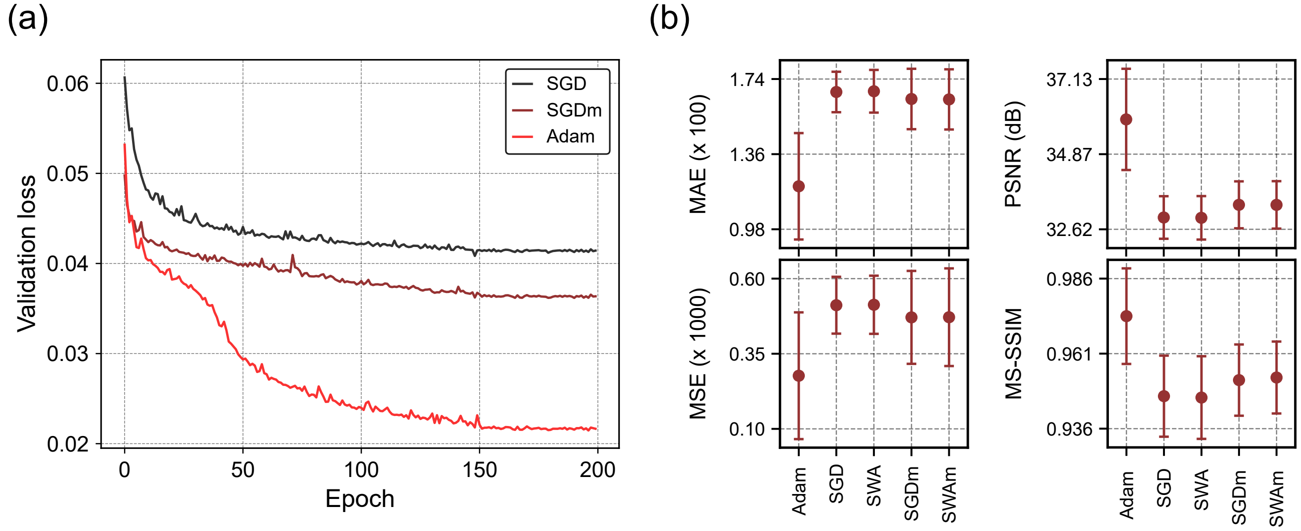

FIG. 5. Denoising performance for different optimizers using the IRUNet architecture. Next to adaptive momentum estimation (Adam), gradient descent methods such as stochastic gradient descent (SGD), and stochastic weight averaging (SWA), both with and without momentum, have been considered. (a) Validation loss curves for Adam and SGD with (m) and without momentum. (b) Mean values (dots) and standard deviation (error bars) of standard image quality metrics obtained after evaluating the trained networks on the separate test set described in the main text.

G. Receptive field of the neural networks

A key property of every convolutional neural network (CNN) is its receptive field, which defines the amount of context information available to the neural network for learning. Ideally, the receptive field should be large enough to capture the features of interest. In our case, these are rod-shaped CDW order that have a spatial extension of up to a third of the larger image dimension, roughly 80 pixels. For a simple single-path network such as VDSR, the receptive field using D consecutive convolutional layers with kernel size 3 and unit stride is given as $(2D{+}1)\times(2D{+}1)$ [6, 7, 15]. In this work, we used 20 convolutional layers resulting in a receptive field of 41×41 pixels. For more complicated

network structures, such as IRUNet, the receptive field cannot be calculated in a straight-forward fashion [15, 16] because it strongly depends on whether, for example, skip connections, non-linear activation functions, and pooling layers are utilized. Using a gradient-based backpropagation method [17] we estimate the receptive field of the used IRUNet network to be around 170×200 pixels, which is much larger than the receptive field of VDSR. Nevertheless, IRUNet does not yield superior results compared to VDSR, suggesting that a larger receptive field does not necessarily relate to a better denoising performance in this context.

H. Background subtraction

As described in the main text, a background subtraction has been performed prior to the line-profile analysis of the charge-density-wave signal. This process involves the summation of pixel intensities within a region-of-interest (ROI) around the signal and subtraction of neighbouring background ROIs. The placement of signal and background ROIs for individual h, k, and ℓ scans is illustrated in Figure 6.

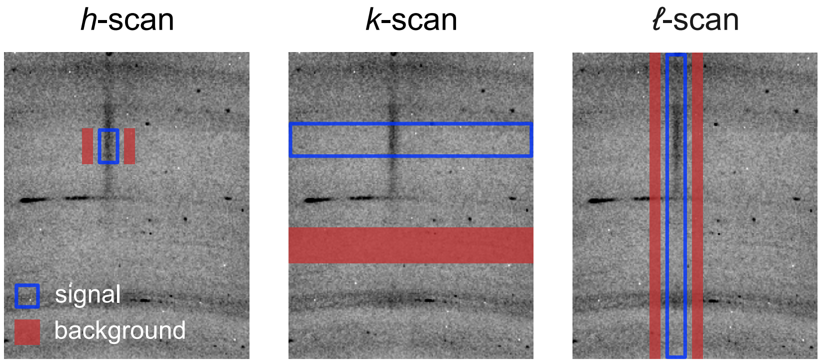

FIG. 6. Placement of signal and background region-of-interest (ROI) for h, k, and ℓ scans. In the case of h and ℓ scans, the background ROI consists of two rectangles of equal sizes, situated next to the signal ROI. The combined size of these two rectangles matches the size of the signal ROI. For k scans, a single rectangle is used as the background instead.

[1] H. Zhao, O. Gallo, I. Frosio, and J. Kautz, IEEE Transactions on Computational Imaging **3**, 47 (2017).
[2] Y. Kim, D. Oh, S. Huh, D. Song, S. Jeong, J. Kwon, M. Kim, D. Kim, H. Ryu, J. Jung, W. Kyung, B. Sohn, S. Lee, J. Hyun, Y. Lee, Y. Kim, and C. Kim, Review of Scientific Instruments **92**, 073901 (2021).
[3] Z. Wang, A. Bovik, H. Sheikh, and E. Simoncelli, IEEE Transactions on Image Processing **13**, 600 (2004).
[4] Z. Wang, E. Simoncelli, and A. Bovik, in *The Thrity-Seventh Asilomar Conference on Signals, Systems & Computers, 2003*, Vol. 2 (2003) pp. 1398–1402.
[5] F. H. Gil Zuluaga, F. Bardozzo, J. I. Rios Patino, and R. Tagliaferri, in *2021 43rd Annual International Conference of the IEEE Engineering in Medicine Biology Society (EMBC)* (2021) pp. 3483–3486, ISSN: 2694-0604.
[6] J. Kim, J. K. Lee, and K. M. Lee, in *2016 IEEE Conference on Computer Vision and Pattern Recognition (CVPR)* (IEEE, 2016) pp. 1646–1654.
[7] K. Zhang, W. Zuo, Y. Chen, D. Meng, and L. Zhang, IEEE Transactions on Image Processing **26**, 3142 (2017).
[8] S. Raschka, arXiv:1811.12808 [cs.LG] (2020).
[9] S. S. Alahmari, D. B. Goldgof, P. R. Mouton, and L. O. Hall, IEEE Access **8**, 211860 (2020).
[10] D. Picard, arXiv:2109.08203 [cs.CV] (2020).
[11] D. P. Kingma and J. Ba, arXiv:1412.6980 [cs.LG] (2017).
[12] A. C. Wilson, R. Roelofs, M. Stern, N. Srebro, and B. Recht, arXiv:1705.08292 [cs, stat] (2018).
[13] P. Zhou, J. Feng, C. Ma, C. Xiong, S. Hoi, and W. E, arXiv:2010.05627 [cs, math, stat] (2020).
[14] P. Izmailov, D. Podoprikhin, T. Garipov, D. Vetrov, and A. G. Wilson, arXiv:1803.05407 [cs, stat] (2019).
[15] A. Araujo, W. Norris, and J. Sim, Distill 10.23915/distill.00021 (2019).
[16] W. Luo, Y. Li, R. Urtasun, and R. Zemel, arXiv:1701.04128 [cs.CV] (2017).
[17] ShelfWise, github.com/shelfwise/receptivefield (2020).
